# Overburden structure fracture evolution and ground pressure behavior under oblique residual coal pillars in thick seam mining

**DOI:** 10.1038/s41598-025-16295-9

**Published:** 2025-08-22

**Authors:** Hongwei Wang, Chengpeng Tian, Yong Liu, Yanjun Li, Meng Lu, Jianqiang Jiao, Jinyuan Bai, Zhijie Qiao

**Affiliations:** 1 College of Energy and Mining Engineering, Xi’an University of Science and Technology, Xi’an, 710054 China; 2 Key Laboratory of Western Mine Exploitation and Hazard Prevention, Ministry of Education, Xi’an, 710054 China; 3 Shaanxi Key Laboratory of Ground Control, Xi’an, 710054 China; 4 Yanbei Coal Mine, Huating Coal and Electric Power Co., Ltd, Huating, 744100 China; 5 Xi’an Research Institute of China Coal Technology Engineering Group Corp, Xi ’an, 710077 China; 6 Cathay Safety Technology Co., Ltd, Beijing, 100012 China

**Keywords:** Thick coal seams, Split-level fully mechanised top coal caving, Residual section coal pillars, Oblique arrangement of the working face, Overburden structure, Engineering, Coal

## Abstract

This study investigates the complex overburden fracture movements and mining pressure evolution induced by obliquely arranged lower-slice extraction passing through residual upper-slice coal pillars in split-level fully mechanized top coal caving of extremely thick coal seams. Through integrated physical simulation, FLAC^3D^ numerical analysis, and field monitoring, the instability mechanisms of residual pillars and their impact on strata behavior were elucidated. The internal and external fields and the evolution characteristics of the overlying strata and structure in the inclined and lower-slice working faces, respectively, were determined. Key findings include: (1) lower-slice extraction induced large-scale overburden collapse through residual pillar instability, with progressive roof structure evolution. (2) When the working face was within 15 m of the section pillar, the maximum vertical stress reached 46.7 MPa (9.9% increase), with significant pillar deformation. When the working face was below the section pillar, a crescent-shaped stress-distribution pattern was observed. (3) Varying obliquely intersecting position between the lower-slice face and the overlying pillar generated a quasi-symmetric trapezoidal fracture pattern. Structural dynamic instability induced an evolutionary sequence of asymmetric double-arch, symmetric double-arch, and single-arch internal-collapse configurations. These findings help extract extremely thick coal seams safely using the split-level fully mechanised top coal caving method.

## Introduction

China maintains proven coal reserves totalling approximately 1.341 trillion tonnes, with thick coal seams representing 44% of its total reserves. Ultra-thick coal seams exceeding 8 m in thickness account for half of these thick seam reserves and are extensively distributed across China’s principal coal-producing regions, including Shandong, Huanglong, and Xinjiang. The output from these ultra-thick seams constitutes approximately 25% of China’s total coal production^1^ and plays a pivotal role in ensuring China’s energy security. With the development of comprehensive mining technology, extra-thick coal seams with thicknesses of more than 20 m have been subjected to stratified comprehensive mining and caving mining methods. The stratified comprehensive caving process has the disadvantages of a high excavation rate, tight production succession, and low working face efficiency. With the development of comprehensive caving mining technology, stratified caving mining technology has become widely used for extra-thick coal seams. However, because of the high mining intensity, the mine pressure in the mining area is severe, the overburden is severely damaged, the fracture zone is abnormally developed, and serious surface damage can occur.

Numerous scholars have conducted extensive research on the key aspects of slice mining in extremely thick coal seams, including overburden structure evolution, strata pressure behaviour, and the influence of residual section coal pillars on subsequent lower-layer extraction. Yu et al.^2–4^ investigated the instability mechanisms of the overburden strata structures in ultra-thick coal seam mining. A structural evolution model for large-scale goaf strata was developed by introducing novel concepts of ‘macro-space structures’ and near-far-field domains to characterise mining-induced strata behaviour. Based on the key strata theory, Dou et al.^5–8^ divided the spatial structure of the overburden in the mining area into three basic categories: ‘O-X’, ‘F’, and ‘T’, and systematically analysed the characteristics of the three structures and the movement morphology of the overburden fracture. The stability of coal seam working faces under repeated mining of close-distance coal seams was analysed by Xiong et al.^9^ It was found that during lower coal seam extraction, two types of ‘arch-shaped’ structures and one ‘arch-beam’ structure were formed in the overlying strata above the stope. Three distinct levels of frequent roof pressure phenomena were identified, demonstrating readily induced working face failure. Through physical similarity simulation and theoretical analysis, the overburden strata movement characteristics in shallow coal seams were investigated by Wang et al.^10^ The results revealed that the key stratum between coal seams was suspended to form a ‘fixed beam’ prior to its failure. After the fracture of the key stratum, the fragmented rock blocks above it synchronously subsided, forming an articulated structure before the eventual collapse. Han et al.^11,12^ simplified the key strata of the curved subsidence zone and solid coal side fracture zone into multi-strata superimposed on infinite and semi-infinite elastic foundation plates and simplified the broken key strata of the side fracture zone of the goaf into multi-strata superimposed on Voussoir beams. Lou et al.^13^ established a beam-arch binary structure model based on the evolution law of mining stress and concluded that, during the mining process, the overburden stratum was broken to form a beam structure in the collapse zone and a stress transfer arch structure in the fracture zone. Zhang^14^ believed that the direct roof characteristics straightforwardly determine the intensity of the mine pressure manifestation in the fully mechanised caving face. They divided the overburden structure of the fully mechanised caving face with a large mining height in the Baode Mine into a Voussoir and short cantilever beam structures through physical simulation combined with theoretical analysis. Results indicated that the cantilever beam structure of the overburden primarily caused the strong mine pressure to manifest in the fully mechanised caving face. Yang^15^ studied the mine pressure law and key technologies of 19 extremely large mining-height working faces through field measurement, theoretical analysis, and physical simulation. They found that as the mining height of the extremely large mining height working face increased significantly, the first sub-key stratum of the overburden was prone to collapse. Moreover, the formation of a Voussoir beam structure was challenging, and a periodic break of the ‘cantilever beam’ structure was observed. With the increase in the burial depth, the breaking of the high-position key strata gradually increased the influence of the mine pressure manifestation on the extremely large mining-height working face, aggravating the mine pressure manifestation in the working face.

Considering the stability of the coal pillar in the remaining section and its influence on the mining of the lower slice, Wang et al.^16^ developed a mechanical model of coal wall behaviour under coupled static–dynamic loading to investigate its response characteristics to transient roof structure instability. Their findings indicated that an abrupt roof failure induced stepwise variations in the static stress response of coal walls. Furthermore, proactive slotting and pressure-relief techniques were proposed as control measures, and the optimal slotting parameters were quantitatively determined. Wang et al.^17^ established a mechanical estimation model for the overall instability and impact induction of a coal body in a double-slice superimposed coal pillar working face, revealing the stress concentration mechanism of the coal pillar and the stress evolution law of the coal pillar. A unique ‘roof-coal pillar’ composite bearing structure was proposed by Wu et al.^18^, and the stress and strength characteristics of each structural unit were rigorously derived. It was demonstrated that structural failure was preferentially initiated in the coal zone adjacent to the lower-seam side interface, ultimately propagating as an oblique penetrating fracture traversing both the coal and roof strata, characterised by a shear-dominated failure mechanism. Huang et al.^19^ studied the overburden structure, pressure law, and advance support pressure evolution law of a working face through a retreat channel and two sections of coal pillars, revealing the stress transfer law of the coal pillar and the dynamic load mechanism of the support. Tian et al.^20^ considered the jagged coal pillars in the first and second mining areas of Shanzhai Coal Mine as the research background, established a lateral support stress estimation model based on the ‘three-band load’ theory, and analysed the stress-distribution characteristics of different areas of the jagged coal pillars. Further, the evolution law of the stress field before and after the formation of the jagged coal pillars was analysed through numerical simulation. Yang et al.^21^ proposed that the dynamic disaster mechanism induced by strong ground pressure from overlying remnant coal pillars occurs through the following process: as the working face advances beyond the coal pillar zone, the pillar-overburden bearing system becomes destabilized due to mining-induced disturbances, resulting in instantaneous energy transfer to the mining area. This energy is rapidly released as kinetic energy, triggering a severe ground pressure dynamic disaster. The energy was instantly transferred to the mining area and released in the form of kinetic energy, causing a strong mine pressure dynamic disaster. Wu et al.^22^ investigated the roof fracture behavior of a fully mechanized longwall face within the stress-concentrated zone beneath coal pillars. Their study quantified the evolution patterns and underlying mechanisms of shield support resistance as the face advanced through the pillar-affected region. The results demonstrated a distinct spatial distribution of support pressures: maximum resistance occurred in the central face section, followed by the lower section, with the upper section exhibiting the lowest values. This phenomenon was attributed to the global rotational subsidence and instability of the overburden’s T-shaped voussoir beam structure.

While previous studies have investigated strata movement in backfilling longwall faces using physical similarity simulation^23^, significant research gaps remain regarding the dynamic fracture evolution mechanisms when inclined working faces traverse residual coal pillars in split-level top-coal caving mining. Existing research has primarily focused on strata behavior in conventional layouts^24^ and control technologies for thick-hard roofs^25^, with fundamental investigations into strata movement laws^26^ and top-coal drawing mechanisms^27^. However, these studies have failed to systematically reveal the asymmetric instability characteristics of pillar-overburden systems under 20° oblique intersection conditions. This study bridges the gap by integrating physical and numerical simulations to establish the first theoretical model of pillar-overburden instability under oblique arrangement mining conditions.

The 5# coal seam in Yanbei Coal Mine adopts an oblique arrangement method between the lower-slice fully mechanized caving face and the upper-slice goaf. As the upper-slice working faces have been fully extracted, the integrity of the overlying strata has been compromised, resulting in various forms of plastic failure and distinct strata pressure manifestations during lower-slice mining. This study takes the 250,203 fully mechanized caving face in the lower-slice of Yanbei Coal Mine as the engineering background. By analysing the overburden movement and stress distribution patterns during fully mechanized caving mining under oblique arrangement with residual section coal pillars left from upper-slice mining, the research reveals the structural evolution characteristics of overlying strata when the lower-slice caving face passes through residual section coal pillars. The findings provide valuable references for achieving safe and efficient extraction in thick coal seams using split-level fully mechanised top coal caving.

## Engineering background

### Mine overview

The Yanbei Coal Mine is located in the central and eastern parts of the Huating Coalfield, with a mine area of 12 km^2^ and recoverable reserves of 362 million tonnes. The main mining 5^#^ coal seam is buried at a depth of 506.7 m, with an average coal thickness of 40.8 m and an inclination of 3–16°. It is a gently inclined, soft, and broken coal seam suitable for large-scale, fully mechanised caving mining. The roof is mainly composed of silty mudstone, with an *f*-*coefficient* of 4.4, which is unstable to medium stable, whereas the bottom is mainly mud-cemented coarse sandstone, with an *f*-*coefficient* of 2.4, poor stability, and easy swelling when exposed to water. The physical and mechanical parameters of the coal strata are listed in Table 1.

**Table 1:** Physical and mechanical parameters of coal and rock.

Rock layers	Thickness/m	Buried depth (m)	Test weight (kg·m^− 3^)	Volume force (kN/m³)	Elastic modulus (MPa)	Internal friction angle (°)
Loess	39.50	39.50	1 600	16.00	300	15
Mudstone, conglomerate	169.30	178.85	2 220	22.20	2 790	38
Sandy mudstone	46.25	225.00	2 220	22.20	2 790	35
Conglomerate	8.47	233.47	2 220	22.20	2 790	35
Silty mudstone	14.77	248.24	2 530	25.30	1 740	28
Mudstone	14.52	262.76	2 530	24.20	1 400	31
Fine sandstone	16.45	279.21	2 420	26.40	4 130	26
Argillaceous siltstone	7.68	286.89	2 640	25.30	1 740	25
Silty mudstone	19.68	306.57	2 530	25.30	1 740	26
Argillaceous siltstone	14.35	320.92	2 530	25.30	1 740	36
Medium-grained sandstone	36.98	356.90	2 530	23.60	5 200	32
3^#^ coal seam	0.88	357.78	1 300	13.00	800	35
Fine sandstone	10.43	368.21	2 640	26.40	4 130	35
Mudstone	2.76	372.42	2 420	24.20	1 400	36
Coarse sandstone	12.14	384.56	2 410	24.10	2 860	25
Fine sandstone	13.85	398.41	2 640	26.40	4 130	35
Silty mudstone	18.75	417.16	2 530	25.30	1 740	35
Fine sandstone	37.66	454.82	2 640	26.40	4 130	25
Siltstone	18.28	473.10	2 530	25.30	1 740	32
Silty mudstone	4.50	477.60	2 530	25.30	1 400	32
5^#^ coal seam	33.48	507.60	1 300	13.00	800	35
Coarse sandstone	9.12	516.72	2 630	23.60	2 860	35

The mine has a designed production capacity of 6.0 Mt/a and adopts an inclined shaft single-level up-and-down mountain development, with a total of eight mining areas. Among them, eight working faces were arranged in the upper slice of the 2502 mining area. The strike longwall inclined sliced fully mechanised caving top coal mining method was adopted, and the roof was managed using the full caving method. The mined upper slice working face and the coal pillar width are 200 m long and 20 m, respectively. The lower slice working face 250,203 is the first mining face of the lower slice of the 2502 mining area, with a length of 300 m, slice height of 15 m, mining height of 4 m, coal caving height of 11 m, and a mining-caving ratio of 1:2.75.

**Fig. 1:**
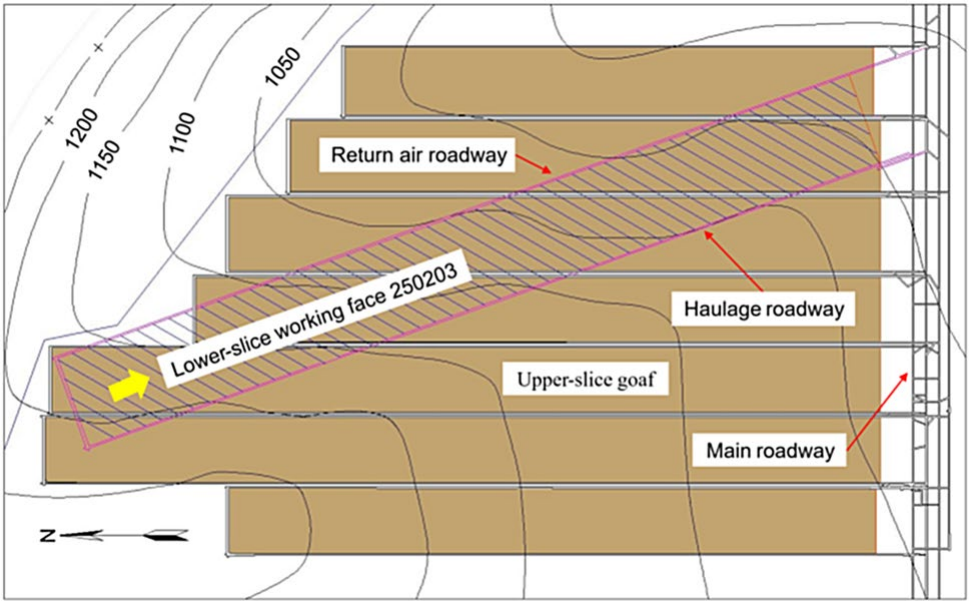
Position relationship between the lower-slice working face 250203 and the upper slice goaf.

The lower slice working face and the coal pillar of the upper-slice residual section were arranged obliquely. During the mining period, the lower-slice working face of 250,203 passed through six upper-slice goafs from 250,207 to 250,202. The positional relationship between the lower slice working face and the upper slice goaf is shown in Fig. 1.

### Layout type of working face under the residual section coal pillar

**Fig. 2:**
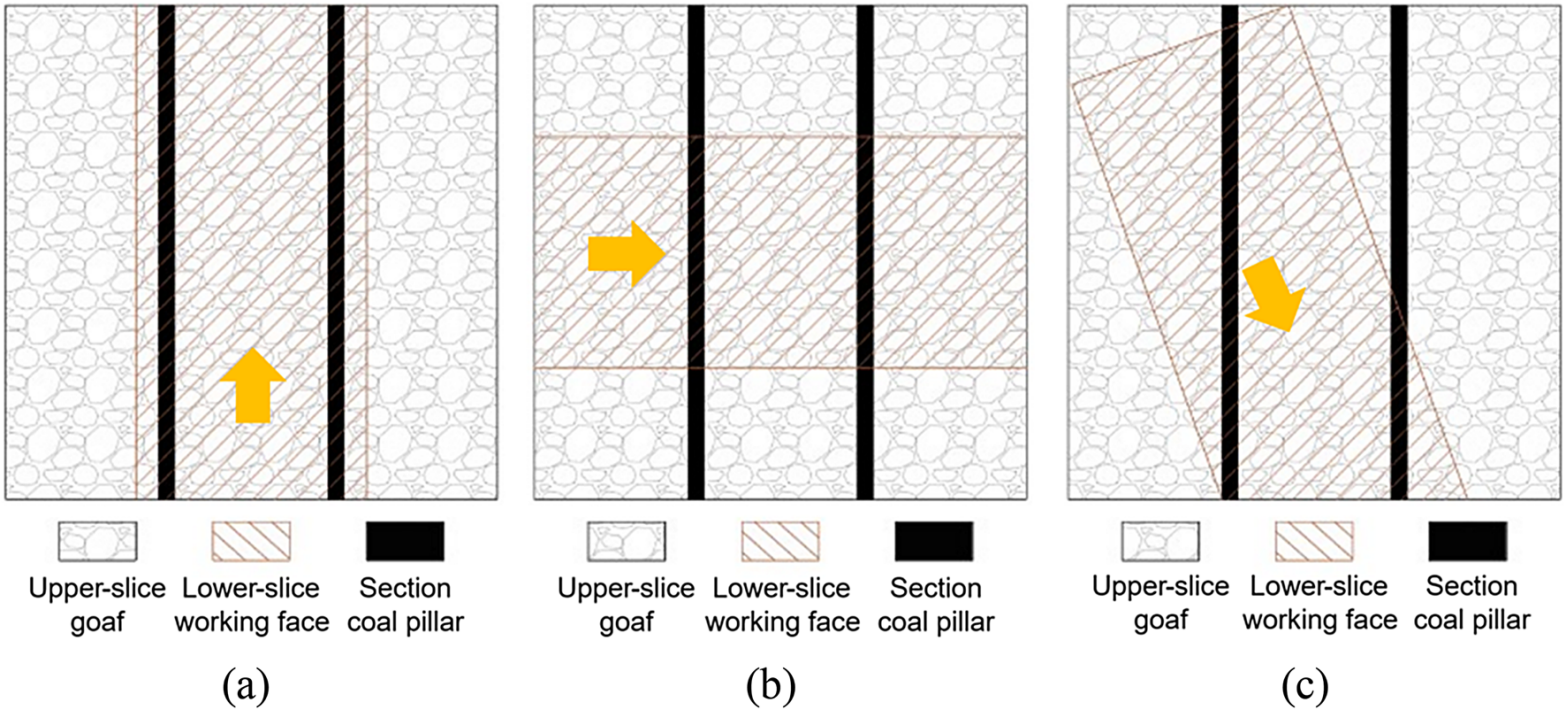
Position relationship between the section coal pillar and the lower slice working face. (**a**) Parallel arrangement. (**b**) Vertical arrangement. (**c**) Oblique arrangement.

According to the overhead projection relationship between the residual section coal pillar of the upper and lower slice working faces, the positional relationship between the two was divided into three layout modes: parallel, vertical, and oblique, as shown in Fig. 2. The parallel layout is the most common working face layout mode, which has little impact on producing the lower slice working face and generally does not cause the problem of strong mine pressure on the working face, but forms an abnormal stress concentration area in the lower-slice coal seam, resulting in difficulties in the maintenance of the mining roadway. In the vertical layout, the entire length of the lower-slice working face entered the influence range of the residual coal pillar of the upper slice simultaneously, causing a violent mine pressure on the working face; however, the duration was short. In the oblique layout, as the lower slice working face advanced along the strike, the upper section of the coal pillar gradually transitioned from one end of the working face to the other when the working face passed through the section coal pillar, causing local and non-fixed pressure on the working face, with a prolonged impact time.

To mitigate the intensity of strata pressure manifestation and prevent the lower-slice working face from remaining entirely beneath the upper-slice residual coal pillars, while simultaneously increasing the face advance length, the 250,203 lower-slice working face was designed with a 20° oblique intersection angle relative to both the upper-slice goaf and the residual section pillars.

## Overburden fracture and migration characteristics of oblique working face

### Design of the physical simulation experiment

To reveal the overburden fracture and migration characteristics when the lower slice working face passes through the coal pillar in the upper slice legacy section, a physical similarity model was established based on the coal and rock slice parameters of the lower slice working face 250,203 at a geometric similarity ratio of 1:300. The model length was 3.0 m, width was 0.2 m, and height was 1.5 m.

#### Similar proportion design

The physical similarity simulation experiment used similar materials to reduce the on-site engineering research object into an experimental model according to the geometric similarity constant based on the field coal and rock strata columnar chart and physical and mechanical parameters. Based on the three laws of similarity, the physical and mechanical parameters of the experimental model were determined: (1) the physical and mechanical parameters of the surrounding rock, namely, bulk density, elastic modulus, cohesion, uniaxial compressive strength, and friction angle; and (2) the size parameters of the surrounding rock, namely, the strike length and vertical height of the coal and rock strata.

Based on a combination of the laboratory experiment platform size and similarity theory, the similarity condition of the model is determined as follows:

1 $${C_l}=\frac{{{l_{\text{p}}}}}{{{l_{\text{m}}}}}=300$$

2 $$C_{\gamma } = \frac{{\gamma _{p} }}{{\gamma _{m} }} = \frac{{2500}}{{1600}} = 1.6$$

3 $${C_\sigma }=\frac{{{\sigma _p}}}{{{\sigma _m}}}={C_\gamma } \cdot {C_l}=480$$

4 $${C_\tau }=\sqrt {{C_l}} =10\sqrt 3$$,

5 $${C_F}=\frac{{{F_{\text{p}}}}}{{{F_{\text{m}}}}}={C_\sigma } \cdot C_{l}^{2}=4.32 \times {10^7}$$

where *C*_*l*_ is the geometric similarity constant; *C*_*γ*_ is the stress similarity constant; *C*_*σ*_ is the stress similarity constant; *C*_*F*_ is the load similarity constant; *C*_*τ*_ is the time similarity constant; *p* is the prototype parameter; and *m* is the model parameter.

#### Similar material selection

Based on the characteristics and mechanical parameters of the coal and rock layers, river sand and pulverised coal were selected as aggregates, gypsum and calcium carbonate were selected as cementing materials, and mica powder was selected as the coal-coal and coal-rock layered material.

#### Similitude model fabrication and monitoring methodology

When fabricating the model, the appropriate materials were initially proportioned by weight according to the lithology of each model stratum. Subsequently, the aggregates and cementing materials were evenly mixed according to the ratios of similar materials. After adding an appropriate amount of water, the mixture was fully stirred and loaded into the model for fabrication. The layered material was evenly distributed after tamping with a heavy object. The physical similarity model ratios are listed in Table 2. The above steps were repeated until model fabrication was completed. Wireless pressure sensors were placed on the floor of 5# coal to measure the support pressure.

**Table 2:** Material ratios for the physical similarity model.

Rock layers	Ratio	Materials required for each slice (kg·cm^− 1^)			
		Sand	Plaster	Calcium carbonate powder	Fly ash
Mudstone, conglomerate	80:3:7	8.53	0.32	0.75	—
Sandy mudstone	80:3:7	8.53	0.32	0.75	—
Conglomerate	80:3:7	8.53	0.32	0.75	—
Silty mudstone	40:2:3	8.53	0.43	0.64	—
Mudstone	45:1:4	8.64	0.19	0.77	—
Fine sandstone	70:3:7	8.40	0.36	0.84	—
Argillaceous siltstone	40:2:3	8.53	0.43	0.64	—
Silty mudstone	40:2:3	8.53	0.43	0.64	—
Argillaceous siltstone	40:2:3	8.53	0.43	0.64	—
Medium-grained sandstone	45:1:4	8.64	0.19	0.77	—
3# coal seam	21:1:2:21	2.69	0.13	0.26	2.69
Fine sandstone	70:3:7	8.40	0.36	0.84	—
Mudstone	45:1:4	8.64	0.19	0.77	—
Coarse sandstone	40:1:4	8.53	0.21	0.85	—
Fine sandstone	70:3:7	8.40	0.36	0.84	—
Silty mudstone	40:2:3	8.53	0.43	0.64	—
Fine sandstone	70:3:7	8.40	0.36	0.84	—
Siltstone	40:2:3	8.53	0.43	0.64	—
Silty mudstone	40:2:3	8.53	0.43	0.64	—
5# coal seam	21:1:2:21	2.69	0.13	0.26	2.69
Coarse sandstone	35:2:3	8.40	0.48	0.72	—

**Fig. 3:**
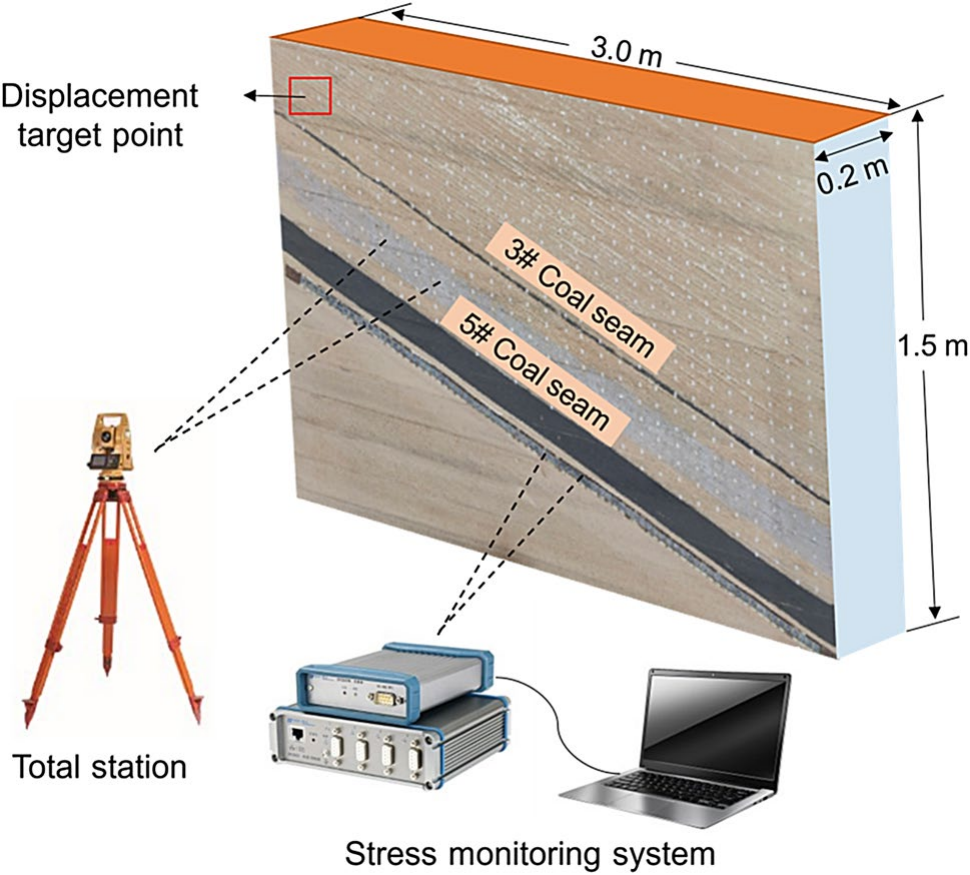
Physical simulation model.

Following the completion of the physical model construction, the displacement monitoring points were systematically arranged on the model surface, and the overburden displacement evolution was subsequently tracked using a total station. After the experiment began, using a 256-channel stress monitoring system, the stress change data during the mining process of the working face were recorded in real time, and the dynamic transmission law of the mining load was analysed. The completed model and monitoring equipment are shown in Fig. 3.

#### Experimental procedures and operational steps

The physical simulation experiment was conducted in strict accordance with the similarity theory and engineering background of the 250,203 lower-slice working face, following the steps outlined below:

##### Model material Preparation

Based on the lithological characteristics of the coal and rock strata (Table 1), raw materials were proportioned by weight according to the ratios specified in Table 2. For example, silty mudstone was prepared using a mixture of river sand, gypsum, and calcium carbonate at a ratio of 40:2:3, while the 5# coal seam was simulated with a blend of pulverized coal, gypsum, calcium carbonate, and fly ash (21:1:2:21). All materials were thoroughly mixed after being weighed proportionally, followed by the addition of purified water and intensive stirring.

##### Layered casting and compaction

The model was constructed in layers corresponding to the actual stratigraphic sequence, with each layer having a thickness of 1 ~ 2 cm (equivalent to 5 ~ 10 m in the prototype, based on the geometric similarity ratio of 1:300). For each layer: The prepared mixture was uniformly spread in the model frame. A 5-kg hammer was used for compaction (3 times per 10 cm²) to ensure consistent density, simulating the natural compaction of strata over geological time. Mica powder was sprinkled between adjacent layers to simulate weak interfaces between coal and rock strata, facilitating accurate observation of interlayer displacement.

##### Installation of monitoring equipment

Stress sensors: Wireless pressure sensors (measurement range: 0–50 MPa, accuracy: ±0.1 MPa) were embedded in the 5# coal seam layer along the strike, to monitor the evolution of support pressure during mining. Displacement monitoring points: Square targets (10 mm × 10 mm in dimension) were affixed to the model surface. These points were tracked using a total station (Leica TS60, accuracy: ±1 mm) to record overburden displacement.

##### Simulation of upper-slice mining

The upper-slice working faces (250203 to 250205) were simulated by sequentially removing the corresponding coal seam layers from the model. Each working face had a length of 67 cm (equivalent to 200 m in the prototype) and a width of 20 cm (equivalent to 20 m in the prototype), consistent with the engineering layout.After mining each upper-slice face, the model was left to stabilize for 24 h to allow for stress redistribution and overburden settlement, mimicking the post-mining consolidation process in the field.

##### Simulation of lower-slice mining and data collection

The 250,203 lower-slice working face (model length: 100 cm, prototype equivalent: 300 m) was simulated through incremental coal seam removal, with each 2 cm advancement in the model representing a 6 m mining progress in the prototype.Displacement data of the surface targets were recorded by the total station.Stress data from the embedded sensors were collected using a 256-channel data acquisition system (sampling frequency: 1 Hz).Visual observations of overburden fractures (e.g., initiation, propagation) were documented using high-resolution photography (Canon EOS R5, 45 MP).

### Overburden rock fracture and migration characteristics in sliced mining

#### Overburden fracture characteristics during upper slice mining

**Fig. 4:**
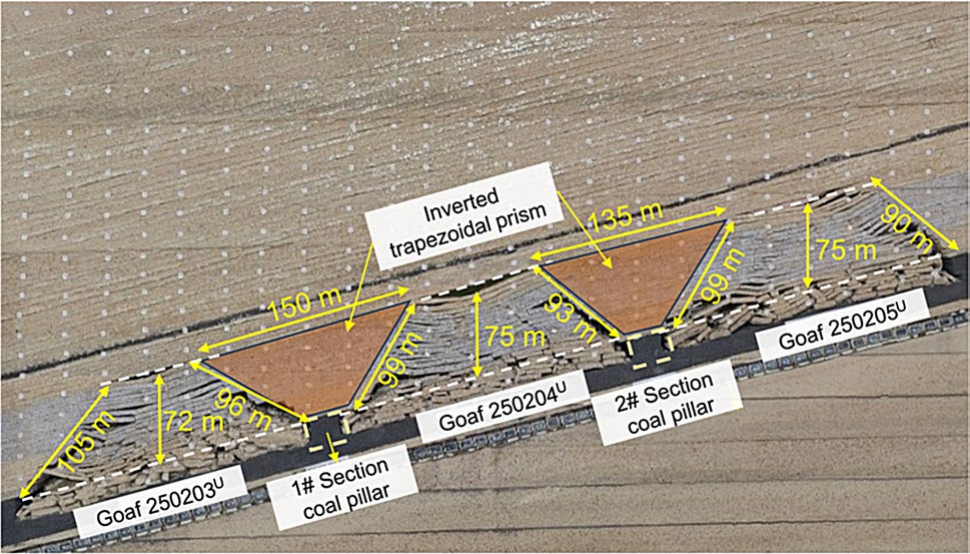
Characteristics of the overburden collapse after mining the upper-slice working face.

**Fig. 5:**
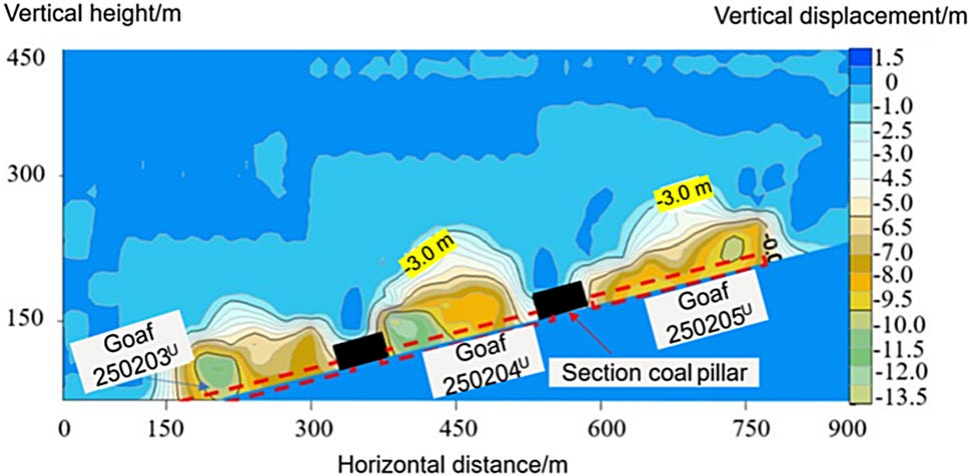
Displacement characteristics of the overlying rock after mining the upper-slice working face.

The collapse characteristics of the overburden tendency and the formation characteristics of the coal pillar section after mining the three working faces in the upper slice are shown in Figs. 4 and 5, respectively. The collapse morphologies of the three working faces in the upper slice after mining have certain similarities, and a trapezoidal goaf area with a narrow top and wide bottom is formed. The collapse heights of upper working faces 250,205, 250,204, and 250,203 were approximately the same— 72, 75, and 75 m, respectively. There were two coal pillars between adjacent working faces. After the overburden collapsed, there was an inverted trapezoidal column area with heights of 72 and 75 m above the section coal pillar, and the upper and lower bases were 150 and 135 m, respectively.

#### Overburden fracture characteristics in lower slice mining

When the lower slice of the working face 250,203 advanced to different positions, the number of upper coal pillars and their positions on the working face exhibited dynamic changes. The different positions of the coal pillars in the section caused significant differences in overlying rock collapse during lower-slice mining.

**Fig. 6:**
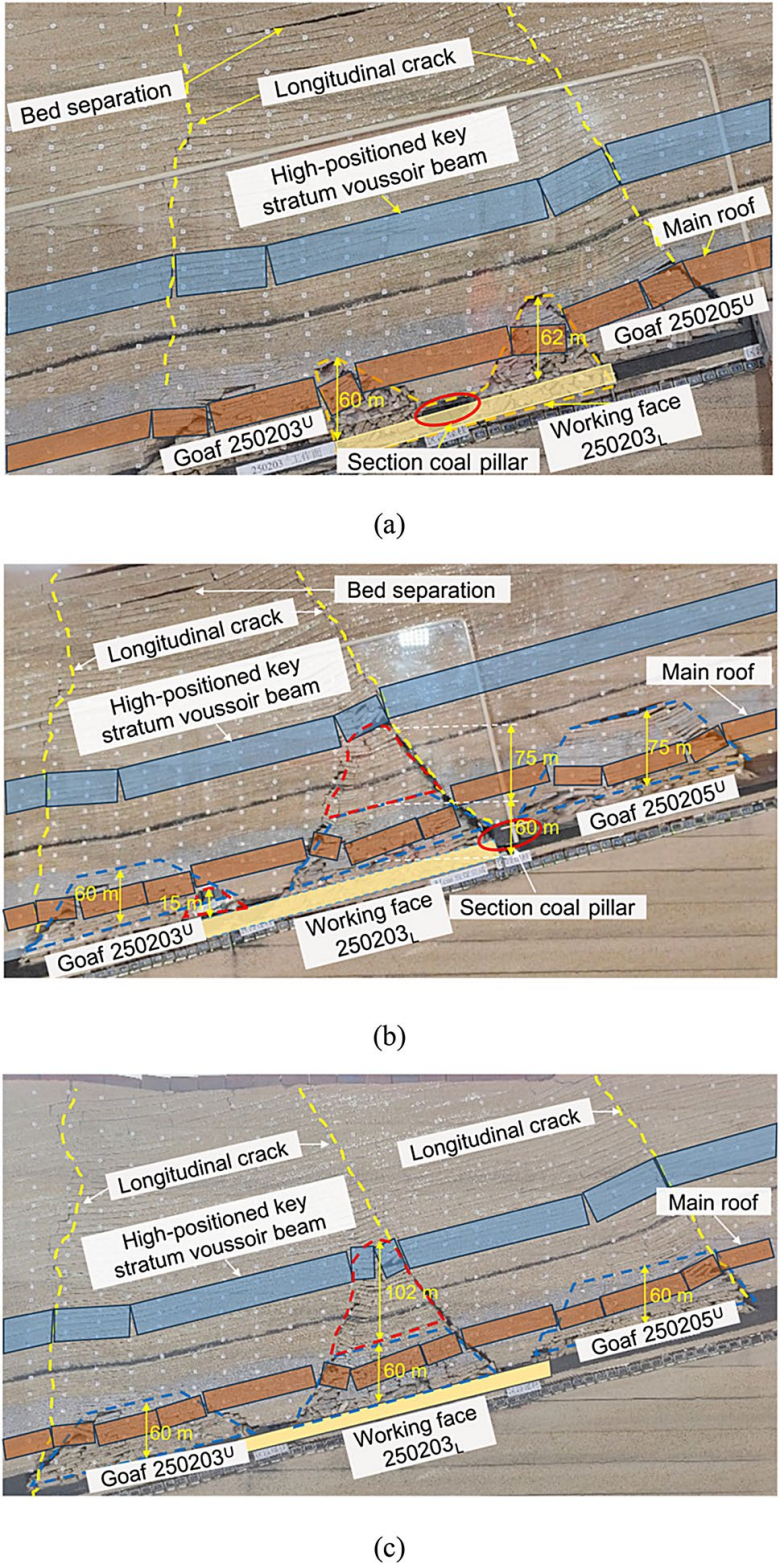
Fracture characteristics of roof strata induced by instability of the section coal pillars at different locations within the working face. (**a**) Coal pillar section in the middle of the working face. (**b**) Coal pillar section on one side of the working face. (**c**) Coal pillar sections on both sides of the working face.

As shown in Fig. 6 (a), a single-section coal pillar, located at the central portion of the lower-slice working face, progressively destabilizes and undergoes shear failure as the ower-slice working face advances. The overburden strata underwent large-scale rotation and sinking ranging from the width of the two upper slice goafs to the top of the model. A longitudinal crack developed on each side, extending from the upper goafs 250,203 and 250,204 to the top of the model. The collapsed overburden in goafs 250,203 and 250,204 was recompacted, and the stacking height was reduced from 72 m to approximately 60 m.

As shown in Fig. 6 (b), a single-section coal pillar located on one side of the lower slice of the working face was unstable and broken. In this case, the instability of the section coal pillar and the main longitudinal crack distribution were similar to those of the single-section coal pillar located in the middle of the working face. The overburden in the goafs of 250,203 and 250,204 disintegrated and collapsed for the second time, forming 135 m and 15 m high collapse zones on both sides of the unstable coal pillar, respectively. The collapse heights of the middle and upper parts of the working face in the lower slice exceeded the height of the original goaf in the upper slice.

As shown in Fig. 6 (c), two-section coal pillars were located on both sides of the lower slice working face. In this case, the instability of the two-section coal pillars affected the three goafs of the upper slices 250,205, 250,204, and 250,203. After the two-section coal pillars became unstable, the overburden collapse exhibited a zoning phenomenon; that is, the high-positioned key strata rock blocks broke. After rotating and sinking, a Voussoir beam structure was formed, which broke to form a Voussoir beam and Voussoir beam combination structure.

**Fig. 7:**
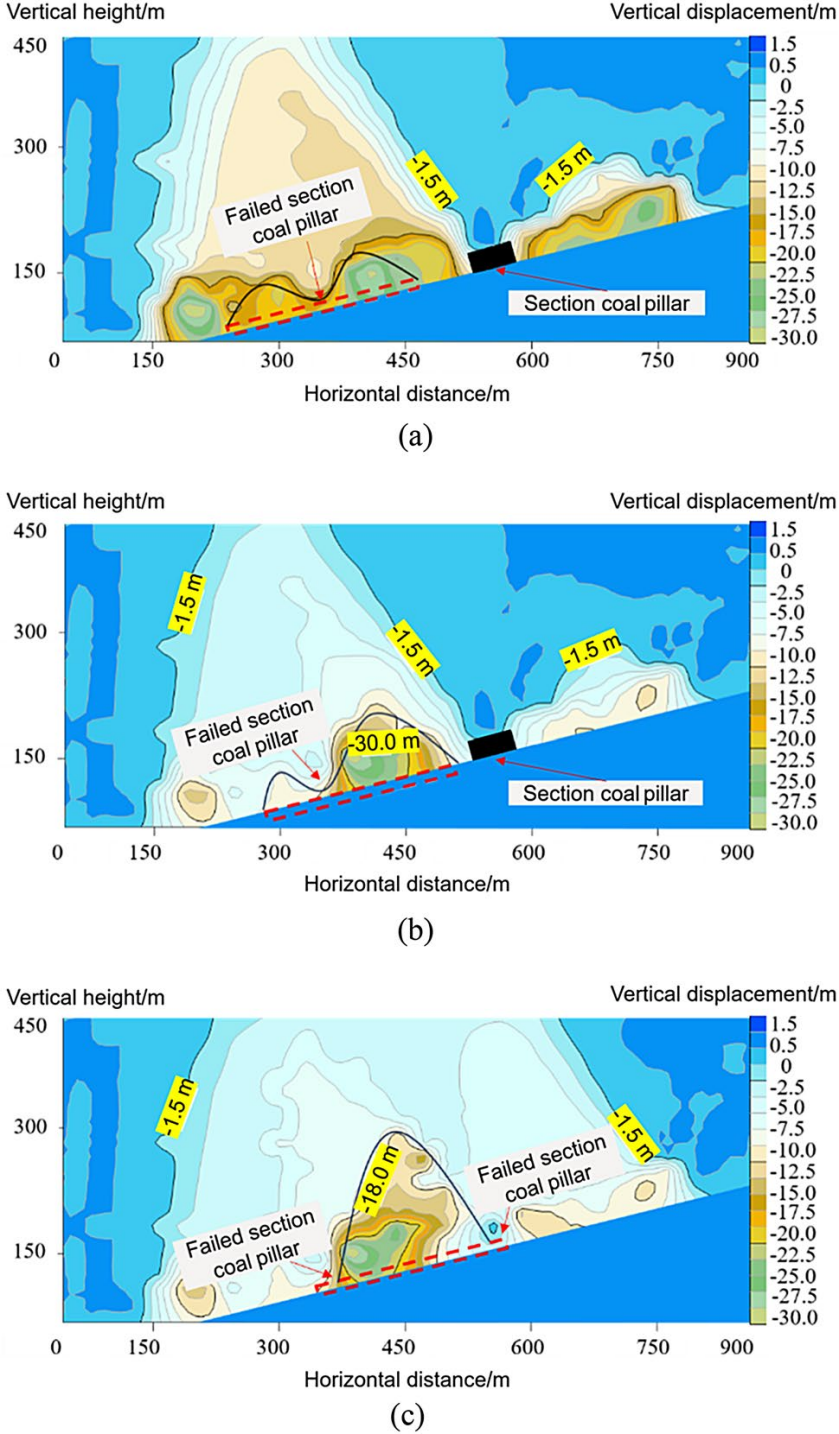
Displacement characteristics of roof strata induced by instability of section coal pillars at different locations within the working face. (**a**) Section of coal pillar located in the middle of the working face. (**b**) Section of coal pillar located on one side of the working face. (**c**) Section of coal pillars located on both sides of the working face.

The displacement characteristics of the overlying strata when the remaining coal pillar sections were unstable are shown in Fig. 7. After the mining of the lower slicing working face led to the instability of the section coal pillars, if a single-section coal pillar was located in the middle of the working face, a “symmetric double-arch” caving zone with a height of 60 m was formed on both sides of the unstable coal pillar, and the vertical displacement ranged from 12.5 to 30 m. When a single-section coal pillar was located on one side of the working face, the lower slicing working face formed two “asymmetric double-arch” caving zones on both sides of the unstable coal pillar, and the vertical displacement ranged from 10.5 to 30 m. When double-section coal pillars were located on both sides of the working face, the lower slicing working face formed a ‘single-arch’ caving zone between the two unstable section coal pillars, and the vertical displacement in this area was 15.0 to 30 m.

**Fig. 8:**
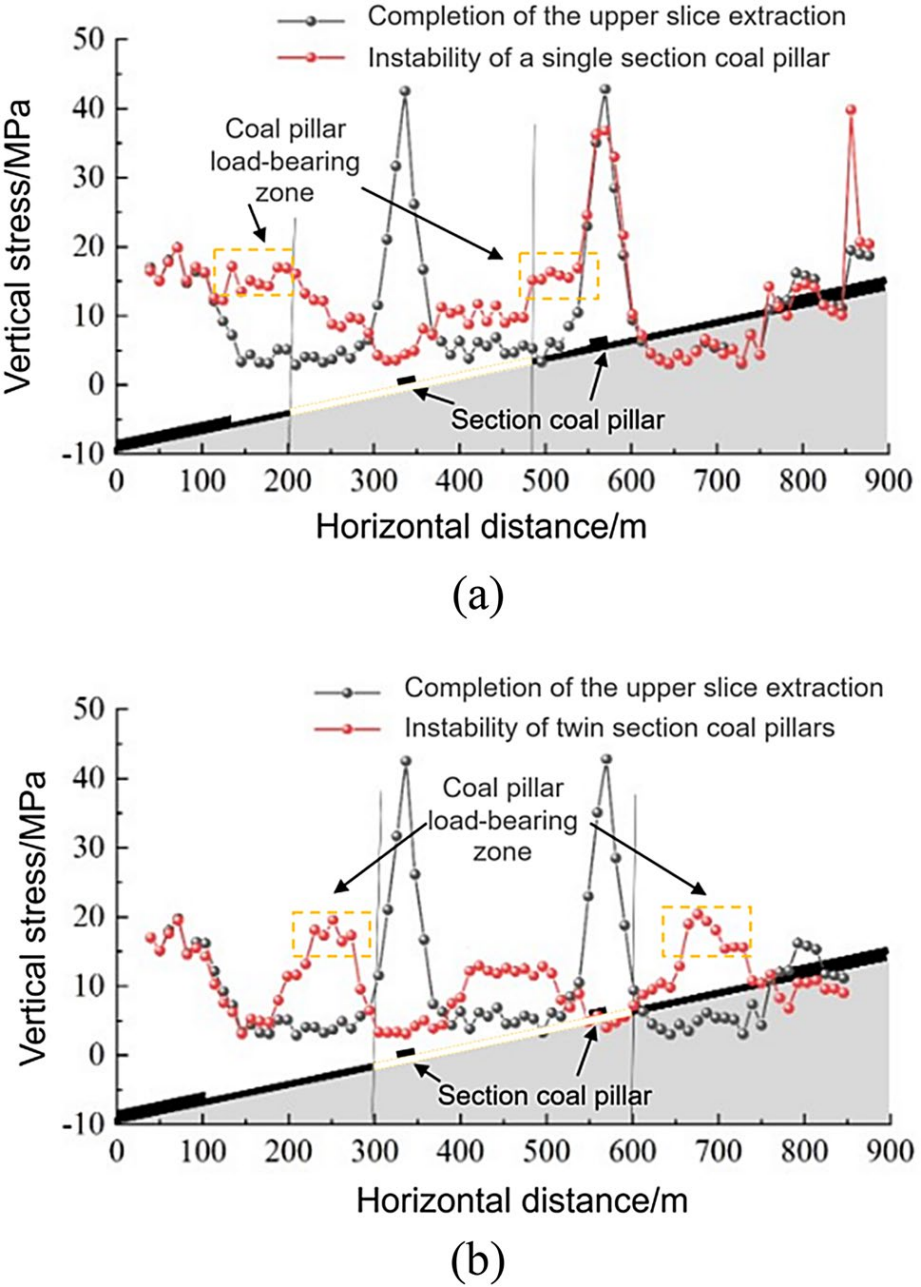
Abutment pressure characteristics of working face for unstable coal pillars. (**a**) Single-section coal pillar above the longwall face. (**b**) Two-section coal pillars concurrently present above the longwall face.

The data from the pressure sensor arranged on the floor shows the measured change characteristics of the working face support pressure when the coal pillar section was unstable, as shown in Fig. 8. When the single-section coal pillar of the working face was unstable, the original support pressure peak disappeared at the section coal pillar, the support pressure on both sides of the section coal pillar increased, the original section coal pillar pressure was transferred to the bearing area on both sides of the section coal pillar, and the support pressure distribution of the floor in the working face was low in the middle and high at both ends; when the double-section coal pillar of the working face was unstable, the original support pressure peak disappeared at the section coal pillar at both ends of the working face, the original section coal pillar pressure was transferred to the bearing area on both sides of the section coal pillar, the support pressure on both sides of the section coal pillar increased, and the support pressure distribution of the floor in the working face was high in the middle and low at both ends.

## Evolution law of overburden structure in oblique working face

### Division of inner and external fields and structural characteristics of overburden rock

To further analyse the evolution law of the overburden structure for the oblique working face of the coal pillar left in the upper slice goaf, the area where the secondary fracture occurs owing to the increase in the mining space of the lower slice working face is defined as the internal field area, and the area where the high overburden stratum is fractured owing to the instability of the section coal pillar is defined as the external field area. However, when the relative positions of the working face and the section coal pillar were different, the collapse form and range of the inner and external field overburdens were also different.

**Fig. 9:**
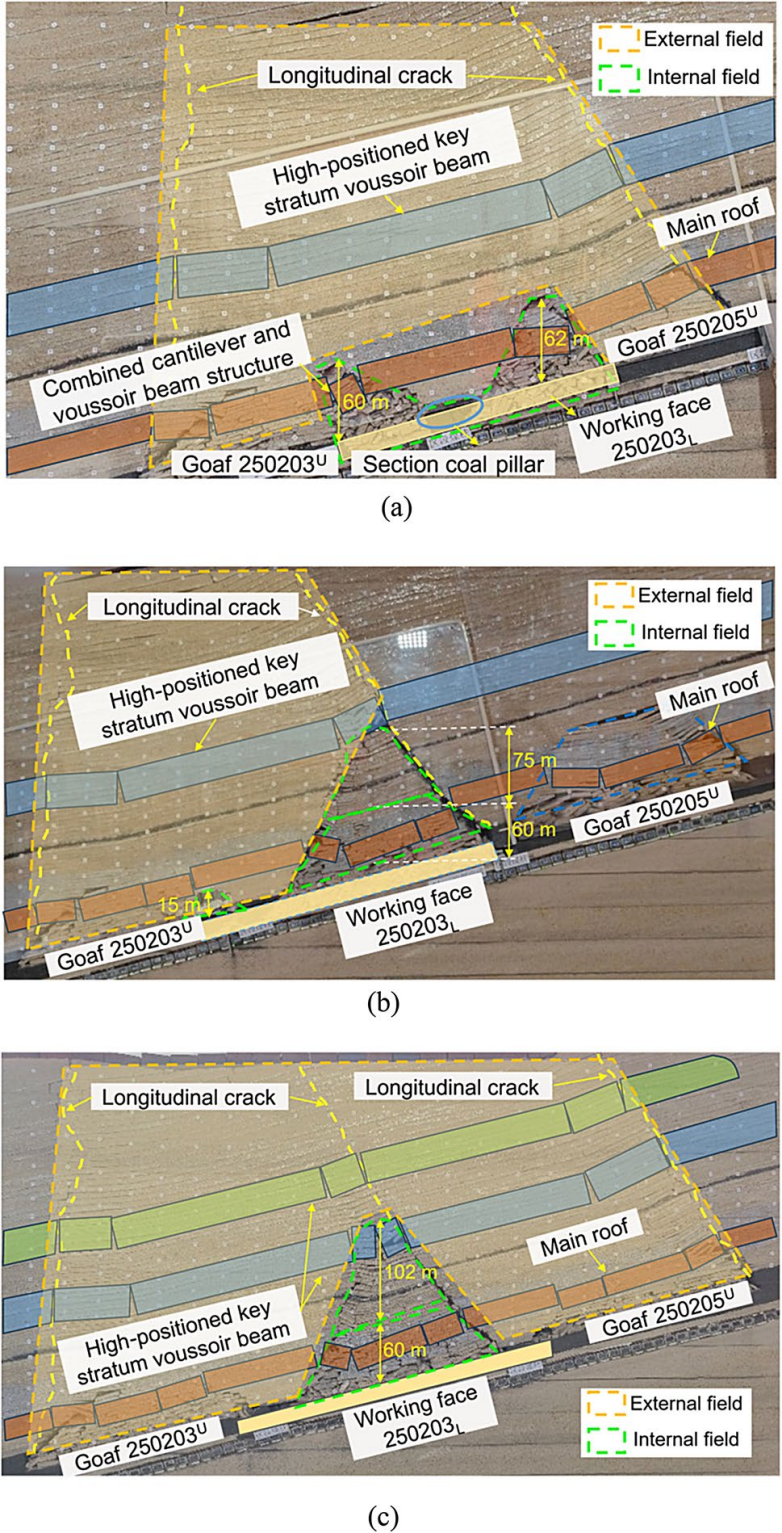
Division of internal and external fields in oblique face mining under the section coal pillar. (**a**) Section of coal pillar located in the middle of the working face. (**b**) Section of coal pillar located on one side of the working face. (c) Section of coal pillars located on both sides of the working face.

When the single-section coal pillar in the middle of the working face was unstable, the height of the lower slice collapse area in the internal field area was 60–62 m, which was composed of collapsed areas on both sides of the coal pillar section, presenting a symmetrical double-arch shape. In the internal field fracture form, the mining of the lower slice oblique working face led to a secondary fracture of the overburden in the upper slice goaf area, and the top rock slice gradually disintegrated and collapsed from bottom to top, forming a cantilever beam and Voussoir beam combined structure; the fracture height was just the height of the collapse area of the original upper slice goaf area. The external field area was trapezoidal, with the bottom width of the two goafs in the upper slice and developed upward to the top of the model, forming a highly positioned Voussoir beam structure on both sides of the external field area, as shown in Fig. 9 (a).

When a single-section coal pillar on one side of the working face became unstable, the lower slice of the internal field area collapsed into two areas with heights of 135 and 15 m. The collapse height increased by 75 m compared to the instability stage of the single-section coal pillar in the middle of the working face, presenting an asymmetric double-arch shape. Owing to the increase in mining space, the upper Voussoir beam structure broke twice, and the collapse range continued to develop upward, with the fracture height reaching below the key strata. The width of the external field area was that of the two goafs in the upper slice, and it developed upward to the top of the model, forming a highly positioned Voussoir beam structure on both sides of the external field area, as shown in Fig. 9 (b).

When the two-section coal pillars on both sides of the working face were unstable, the height of the lower slice collapse area in the internal field area was 162 m, which was 102 m higher than that of the single-section coal pillar instability stage in the middle of the working face. It was in the form of a single-arch with two damaged coal pillars on both sides of the arch. The internal field fracture was a secondary fracture of the gangue rock blocks in the upper slice goaf, forming a cantilever beam and Voussoir beam combined structure. The fracture height exceeded that of the original upper slice collapse area. As the mining space in the excess area increased, the upper key strata broke, causing the collapse range to continue to expand upward. The width of the external field area was the width of the three goafs in the upper slice, and it developed upward to the top of the model, forming a highly positioned Voussoir beam structure on both sides of the external field area, as shown in Fig. 9 (c).

### Evolution law of internal and external field structures of overburden rocks

The positional relationship between the oblique working face of the lower slice and the coal pillars in the legacy section exhibited a dynamic change. Based on the different positional relationships between the oblique working face of the lower slice and the coal pillars in the legacy section, the collapse characteristics of the lower slice mining were analysed.

**Fig. 10:**
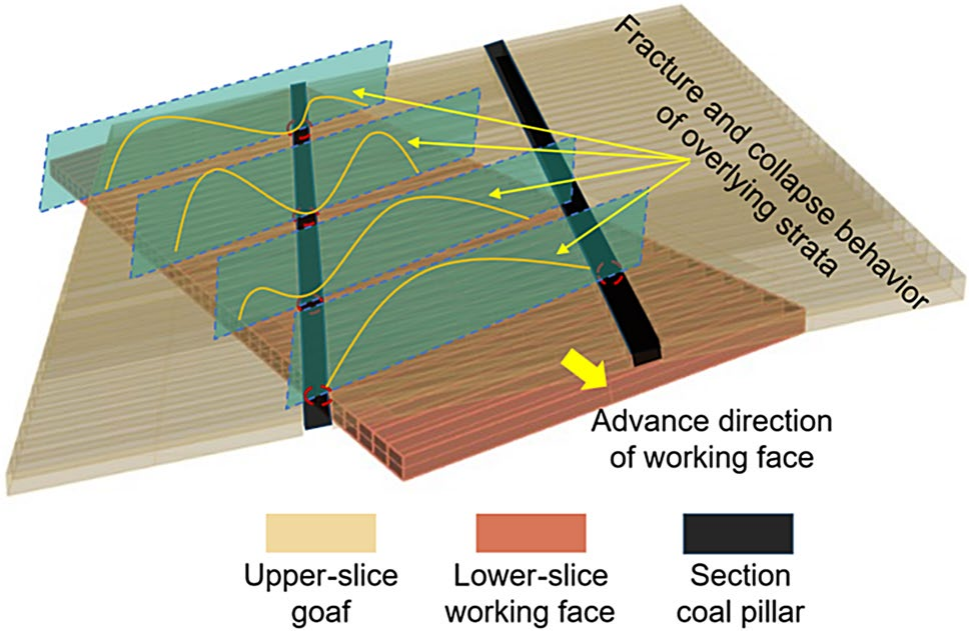
Caving characteristics of the overlying rock in an internal field when coal pillar is located at different positions of working face. Figure 10 was self-drawn by Chengpeng Tian (corresponding author) using Microsoft PowerPoint(Version:2019, https://www.microsoft.com/zh-cn/microsoft-365/powerpoint ).

When the section coal pillar was located in the upper area of the lower slice working face, the collapse morphology was an asymmetric ‘double-arch’ type, and the collapse arch in the lower area was higher than the upper part. When the section coal pillar moved to the lower area of the working face and was located in the middle of the working face, the collapse morphology was a symmetrical ‘double-arch’ type, and the two arch heights were equal. When the section coal pillar was located in the lower area of the lower slice working face, the collapse morphology was an asymmetric ‘double-arch’ type, in which the collapse arch in the upper area was higher than the lower part. When there were two-section coal pillars in the lower slice working face, the collapse morphology was a ‘single-arch’ type, and the collapse arch was higher than the collapse arch of the above-mentioned single-section coal pillar. The changes in the collapse morphological characteristics of the internal field overburden when the residual section coal pillar is located at different positions of the working face are shown in Fig. 10.

It can be deduced from the above analysis that the mining of extremely thick coal seams forms a large-space mining area, and the roof structure in the disturbance zone gradually evolves to form a combined structure of a lower-positioned inverted step-combined cantilever beam and a highly positioned large-structure Voussoir beam. In the single-section coal pillar instability stage, the maximum width of the external field is two goaf widths, and the internal field moves within the external field trapezoidal range with the different positions of the section coal pillars, varying between double-arch and single-arch forms. In the two-stage coal pillar instability stage, the maximum width of the external field is three goaf widths, and the internal field is in the middle of the external field trapezoid, showing a single-arch shape.

**Fig. 11:**
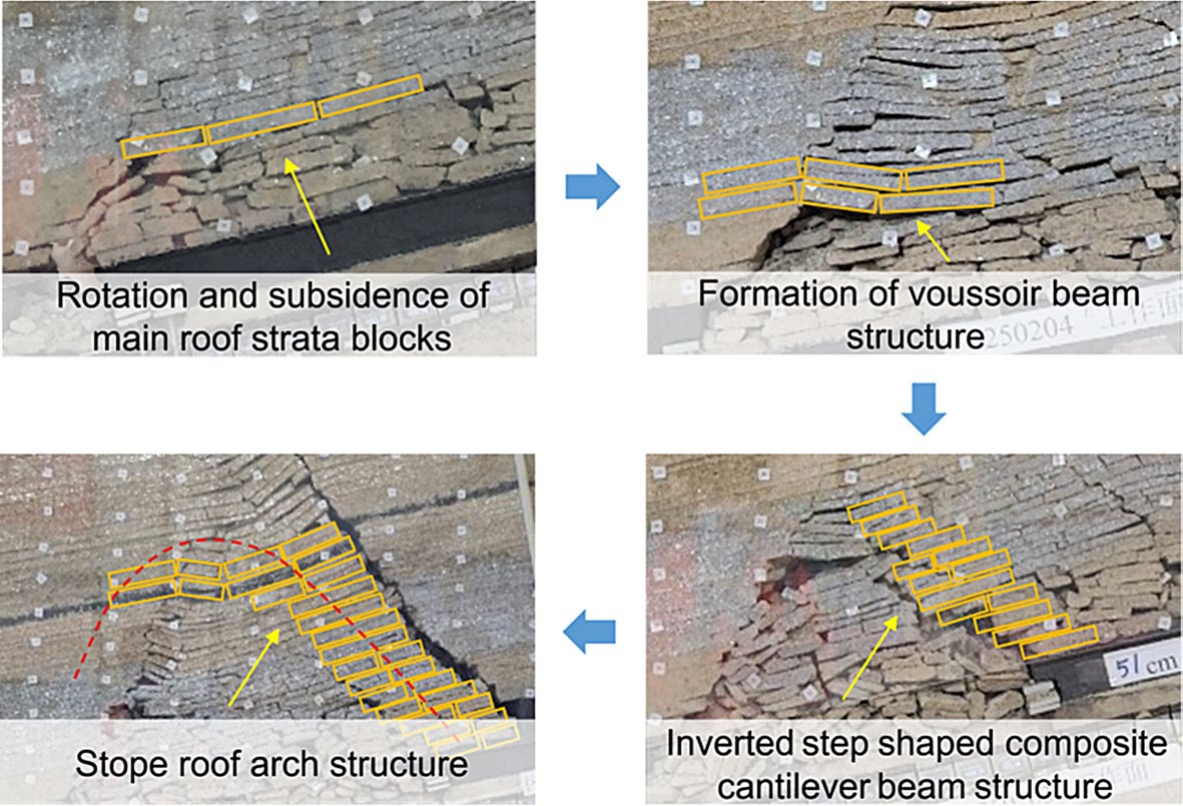
Evolution of the roof structure.

**Fig. 12:**
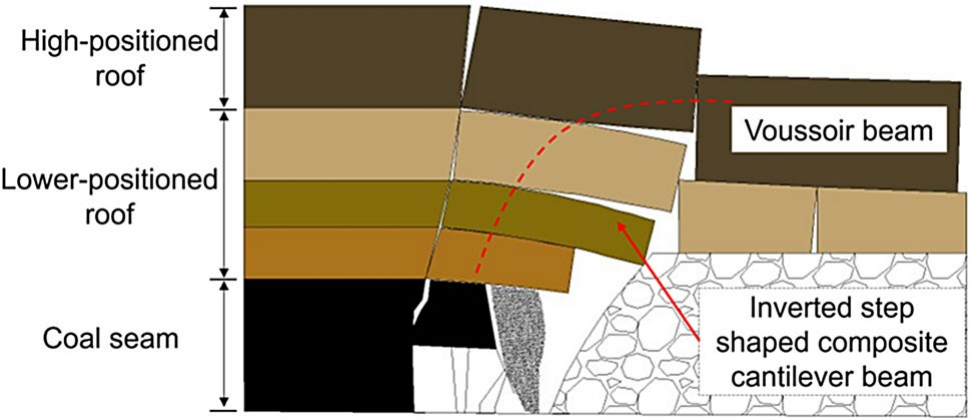
Stope roof arch structure.

The evolution of the overburden strata structure is illustrated in Fig. 11. During the upper slice mining process, the roof structure of the working face consisted mainly of a combination of cantilever and Voussoir beam structures. During the mining process of the oblique working face of the lower slice, the instability of the coal pillar section caused the overburden to sink, causing the broken basic roof rock blocks to rotate, and the Voussoir beam structure was closely arranged in the goaf. After the basic roof rock blocks were broken for the second time, the Voussoir beam structure of the roof of the working face collapsed due to the release of the lower space to form an inverted step-shaped ‘cantilever beam’ structure, and multiple cantilever beams were combined to form an arch structure that played a major bearing role. As the working face continued to advance, the broken rock blocks of the basic roof rotated and sank. The inverted step-shaped cantilever beam structure gradually became unstable from bottom to top, forming loose blocks and collapsing, and the outermost cantilever beam structure piled up to form a larger arch structure. The evolution of the overburden structure is illustrated in Fig. 12.

## Distribution characteristics of mining stress in oblique working face under coal pillar in the remaining section

### Numerical calculation model establishment

Based on the engineering background of the 250,203 lower-slice working face in Yanbei Coal Mine, FLAC^3D^ was used to simulate the stress evolution and surrounding rock failure characteristics during lower-slice mining obliquely under residual upper-slice pillars, aiming to quantify the interaction between the working face and residual coal pillars.

#### Model parameter settings

The model was set to 800 m (length) × 900 m (width) × 500 m (height) to cover the entire mining influence range of the 2502 mining area, avoiding boundary effects on the target area. 16 geological layers were included, consistent with field stratigraphy (Table 1). The 5# coal seam (target seam) had a thickness of 33.48 m and an average buried depth of 507.6 m, with overlying strata dominated by silty mudstone and sandstone, and the floor consisting of weakly water-sensitive coarse sandstone. Derived from laboratory tests (Table 1), e.g., 5# coal seam: bulk density 1300 kg/m³, elastic modulus 800 MPa, internal friction angle 35°; silty mudstone: bulk density 2530 kg/m³, elastic modulus 1740 MPa, internal friction angle 28°.

Mohr-Coulomb model was adopted to describe plastic failure, with large-deformation mode enabled to capture significant strata movement.Upper-slice goafs were filled with weak materials (bulk modulus 1.2 GPa, shear modulus 0.5 GPa) to simulate gangue compaction, calibrated against field settlement data.

#### Boundary conditions and initial conditions

A vertical stress of 3.0 MPa was applied to the top surface (equivalent to overburden weight), and horizontal stresses of 1.5 MPa (50% of vertical stress) were applied laterally, consistent with in-situ measurements in the Huating Coalfield. The bottom boundary was fixed vertically and horizontally; lateral boundaries were fixed horizontally but allowed vertical movement to ensure free deformation.

**Fig. 13:**
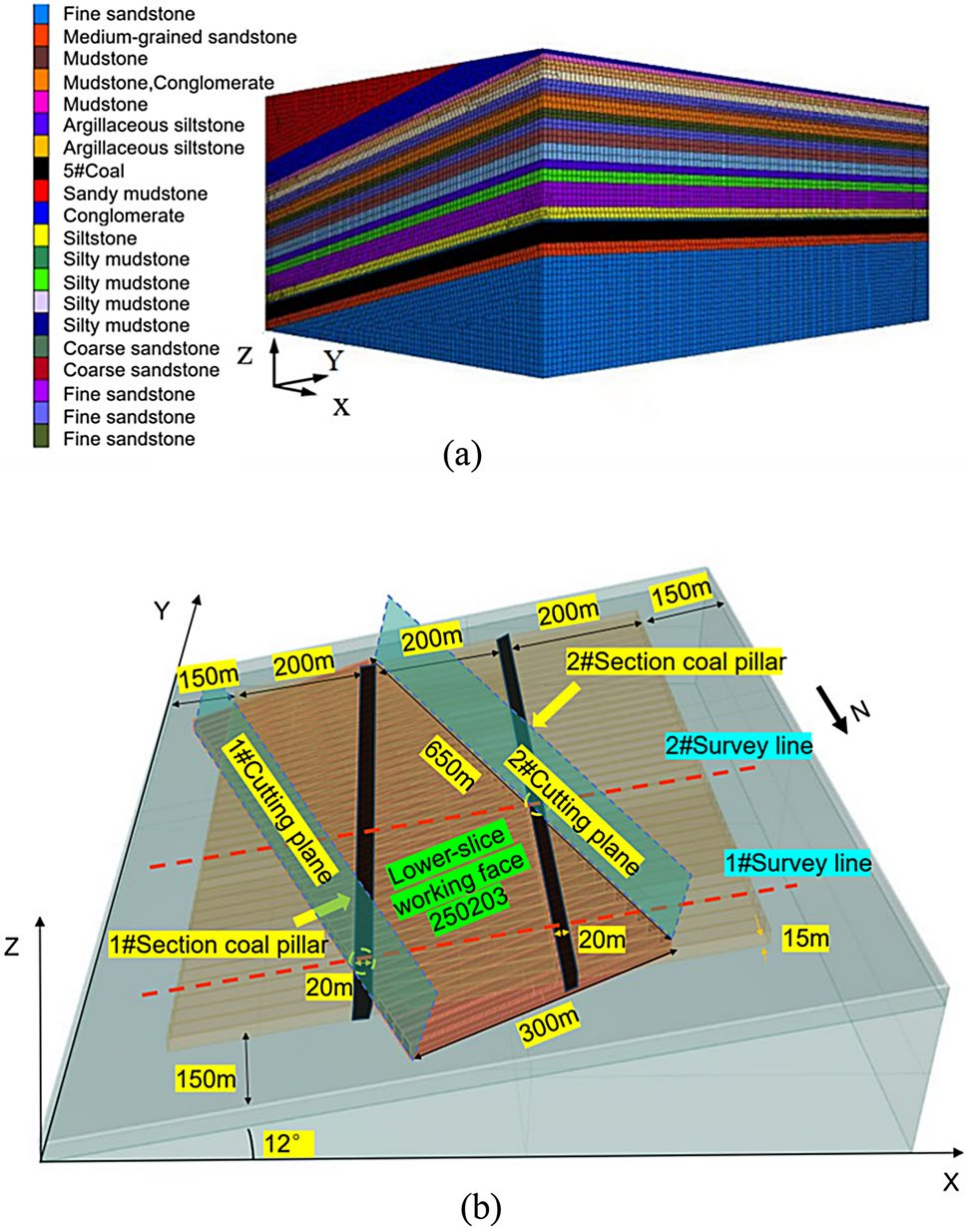
Numerical calculation model. (**a**) Three-dimensional model. (**b**) Schematic of the working face slices. (**a**) was generated by Chengpeng Tian (corresponding author) via FLAC3D software(Version:6.0, https://www.itascasoftware.com/software/flac3d/).

Generated by gravitational loading, balanced until the unbalanced force ratio < 1 × 10^− 5^, matching in-situ stress distribution (vertical stress gradient ~ 0.025 MPa/m). The 250,203 lower-slice working face (length 300 m, mining height 4 m, caving height 11 m) advanced obliquely at 20° relative to upper-slice residual pillars (width 20 m), replicating the actual spatial relationship (Fig. 13a).

#### Simulation steps

Constructed with hexahedral elements (2.4 million total), with refined meshes (2 m×2 m×1 m) in the 5# coal seam and adjacent strata, and coarser meshes (5 m×5 m×3 m) for distant strata.

Upper-slice mining simulation: Working faces 250,203–250,205 were mined sequentially (each 200 m long), with goafs filled with weak materials. The model was balanced after each mining step to simulate stress redistribution.

The 250,203 lower-slice working face advanced obliquely in 50 m steps (equivalent to 5 days of field advancement). After each step, parameters (vertical stress, displacement, plastic zones) were recorded at monitoring points (Fig. 13b). The simulation continued until the working face passed all six upper-slice goafs (total advancement 550 m).

### Stress evolution characteristics of overburden rock above Goaf

**Fig. 14:**
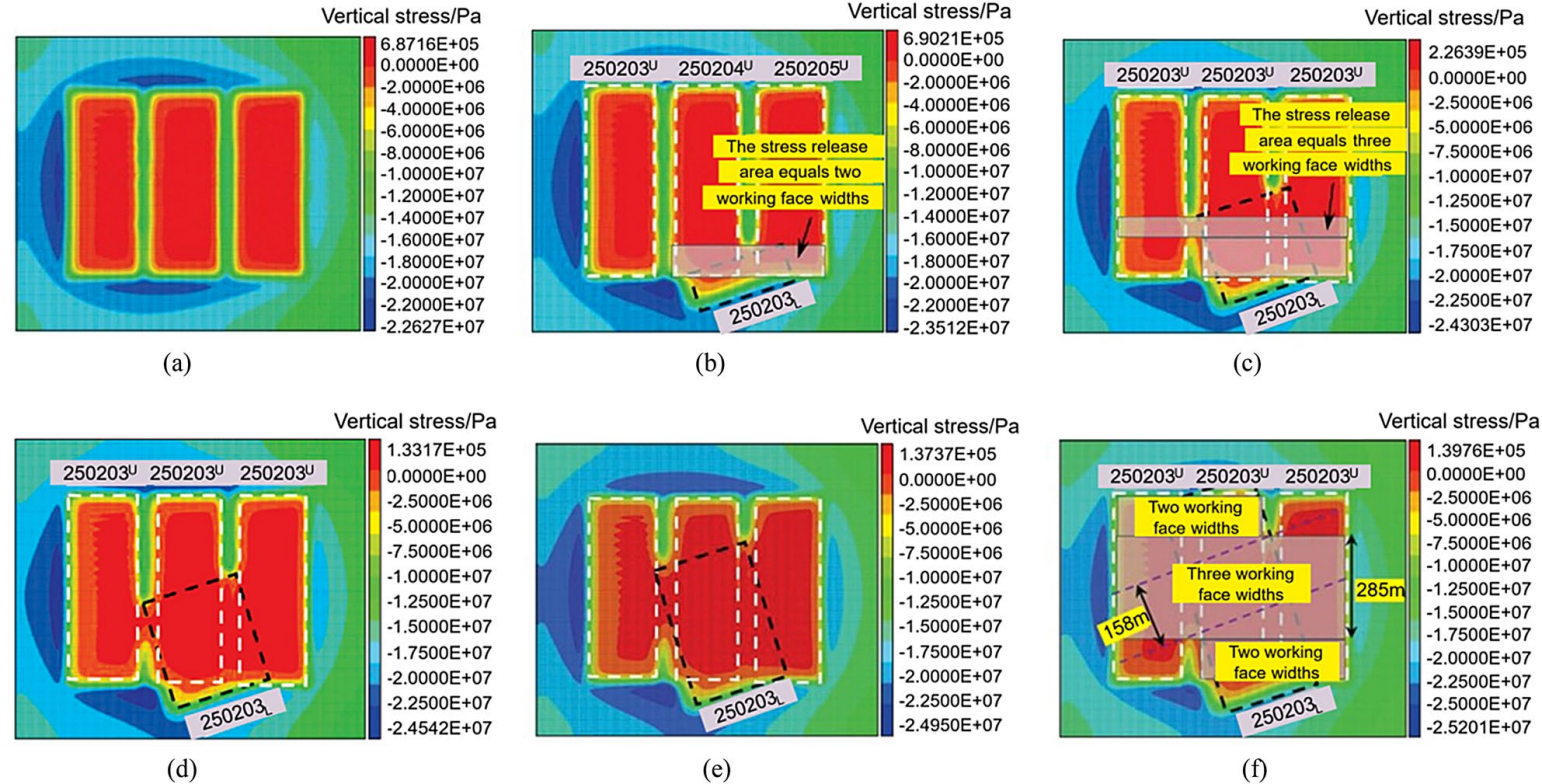
Vertical stress characteristics of the surrounding rock when advancing the lower working face. (**a**) Before mining the lower slice. (**b**) Working face advanced to 100 m. (**c**) Working face advanced to 250 m. (**d**) Working face advanced to 300 m. (**e**) Working face advanced to 400 m. (**f**) Working face advanced to 550 m.

A top-down view was created above the working face. The vertical stress change characteristics of the surrounding rock during the mining process of the lower slice working face are shown in Fig. 14 below: Fig. 14 (which) shows the vertical stress-distribution characteristics of the roof after upper slice mining is completed. Three stress release zones were formed above the working face. Stress concentration occurred above the coal pillar section, and the average vertical stress was 12.87 MPa. During the advancement of the lower slice working face, the lower slice working face passed under section No. 2 of the coal pillar. After the coal pillar section lost its stability, the stress was redistributed. The top stress above the coal pillar was released, and the two stress release areas at the original 250,205 upper and 250,204 upper goaf positions were connected, as shown in Figs. 14 (b) and (c). When the working face advanced to 250–350 m, there were two coal pillar sections, Nos. 1 and 2, on the roof of the working face simultaneously. After the coal pillar lost its stability, the top stress was released. At this time, the original three stress release areas were connected to each other, as shown in Figs. 14 (d)–(f). After the working face advanced to 400 m, it gradually left the No. 2 section coal pillar, and only the No. 1 section coal pillar remained in the working face. The instability of the coal pillar caused the area connected to the roof stress release zone to transform into the original 250,204 and 250,203 upper goaf areas.

### Stress evolution characteristics during entry into and exit from residual section coal pillars in lower slice mining operations

To study the vertical stress changes when the lower slice working face enters and exits the coal pillar, cutting planes 1 and 2 were made parallel to the mining direction of the working face at the coal pillar entering Sect. 2 and exiting Sect. 1, as shown in Fig. 13 (b).

**Fig. 15:**
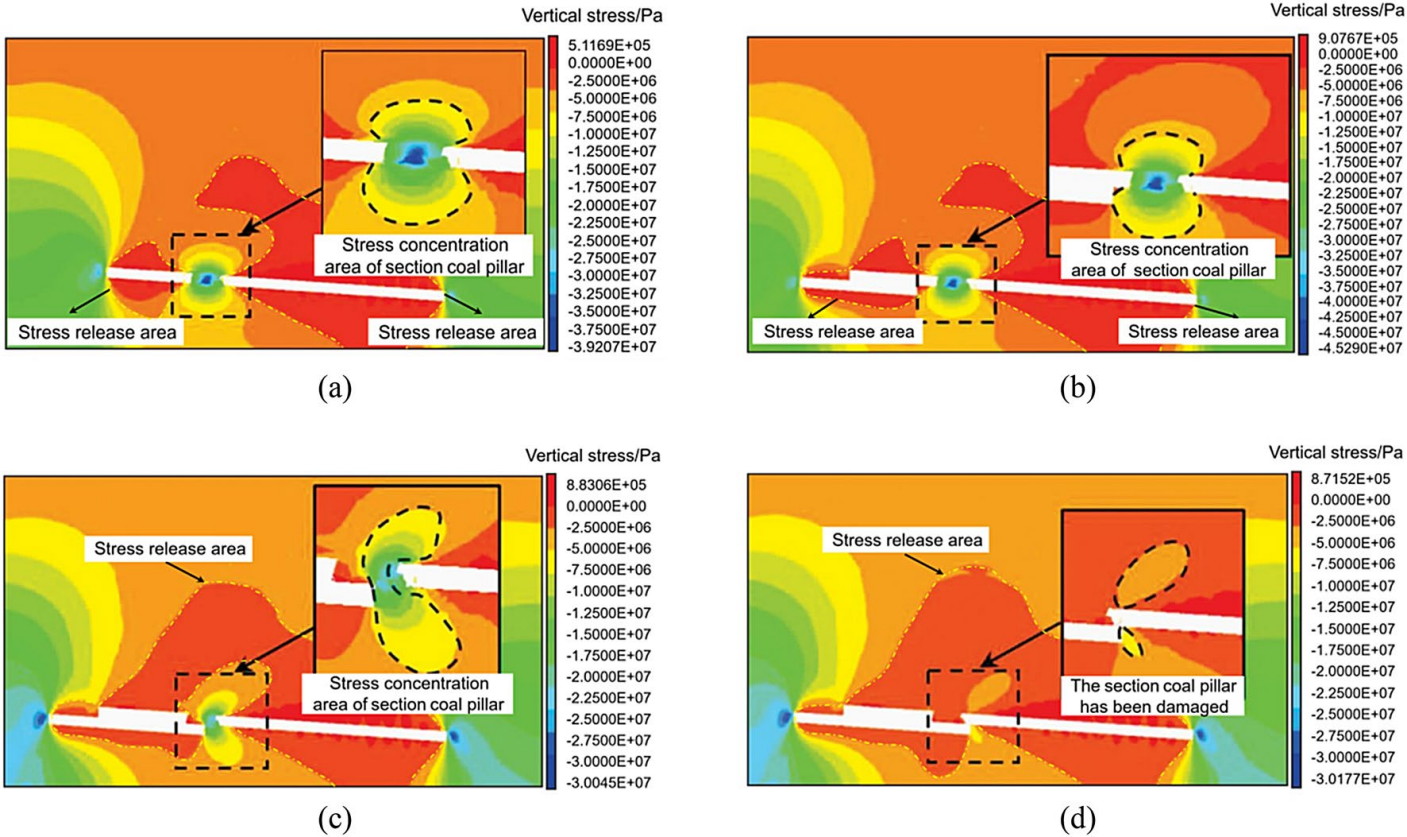
Vertical stress nephogram of the surrounding rock when a coal pillar enters the lower stratified face. (**a**) Before mining the lower slice. (**b**) When the working face is 45 m from the centre of the section coal pillar. (**c**) When the working face is below the centre of the section coal pillar. (**d**) When the working face is 30 m from the centre of section coal pillar.

The characteristics of the vertical stress change in the surrounding rock when the lower slice working face enters the coal pillar section are shown in Fig. 15. When the lower slice was not mined, the vertical stress concentration of the coal pillar section was approximately symmetrical from top to bottom, and the maximum vertical stress was 39.2 MPa. As the distance between the working face and the centre of the coal pillar decreased, the maximum vertical stress of the section coal pillar increased, reaching a maximum value of 45.3 MPa at 45 m from the centre of the coal pillar, which was 13.7% higher than when the lower slice was not mined. When located directly below the coal pillar, the maximum vertical stress of the coal pillar section decreased to 20.0 MPa, and the stress concentration area was distributed in a crescent shape. As the working face continued to advance, the crescent-shaped stress concentration phenomenon gradually disappeared.

**Fig. 16:**
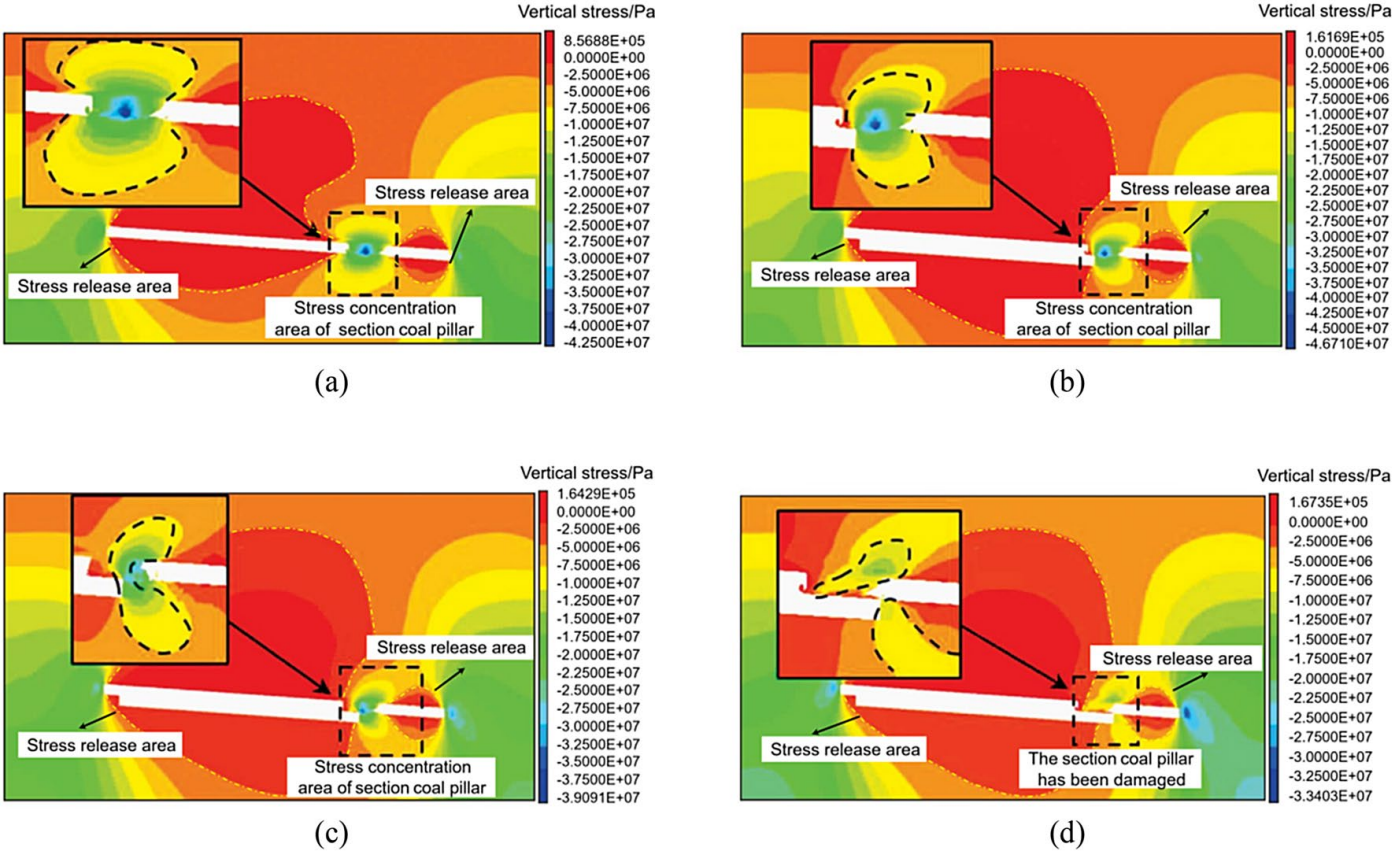
Vertical stress nephogram of the surrounding rock when a coal pillar exits the lower stratified face. (**a**) Before mining the lower slice. (**b**) When the working face is 15 m from the centre of the section coal pillar. (**c**) When the working face is below the centre of the section coal pillar. (**d**) When the working face is 30 m from the centre of section coal pillar.

The characteristics of the vertical stress change in the surrounding rock when the lower slice working face exits the coal pillar section are shown in Fig. 16. When the lower slice was not mined, the vertical stress concentration of the coal pillar section was approximately symmetrical, and the maximum vertical stress was 42.5 MPa. As the working face approached the centre of the coal pillar, the maximum vertical stress increased. When the working face was 15 m away from the centre of the coal pillar, the section coal pillar reached maximum vertical stress of 46.7 MPa, which was 9.9% higher than that when the lower slice was not mined. The coal pillar deformation phenomenon was more obvious. When it was directly below the coal pillar, the maximum vertical stress of the coal pillar section was reduced to 39.0 MPa, and the stress concentration area was approximately crescent-shaped. As the working face continued to advance, the crescent-shaped stress concentration phenomenon gradually disappeared. When the working face passed the centre of the coal pillar by 30 m, the coal pillar section was destroyed, and the crescent-shaped stress concentration area disappeared.

**Fig. 17:**
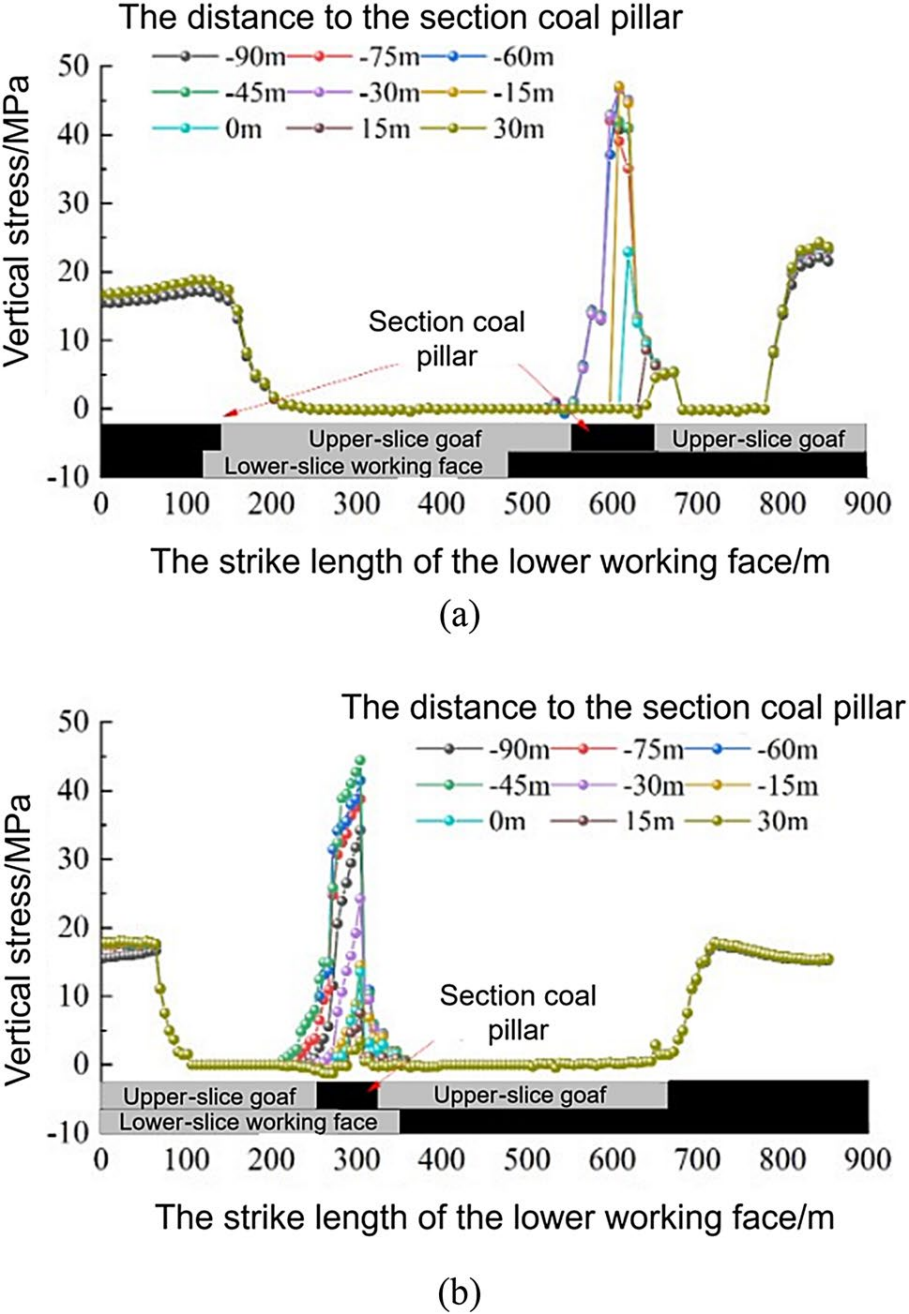
Vertical stress characteristics of the roadway when a coal pillar enters and exits the lower working face. (**a**) Vertical stress characteristics of the haulage roadway. (**b**) Vertical stress characteristics of the return air roadway.

According to the results of the numerical calculation, the vertical stress data of measuring lines 1 and 2 and the vertical stress characteristics of the roadway when entering and exiting the coal pillar section of the lower slice working face are as shown in Fig. 17. The vertical stress of the haulage roadway reached the maximum value of 47.1 MPa when it was 15 m away from the section coal pillar; the vertical stress of the return air roadway reached the maximum value of 44.5 MPa when it was 45 m away from the section coal pillar.

## Discussion

Based on the mining engineering background of the 250,203 lower slice fully mechanised top coal caving face in Yanbei Coal Mine operated by Huating Coal Group, this study elucidates the instability characteristics of upper slice residual section coal pillars and the variation laws of surrounding rock abutment pressure under mining-induced disturbances in lower slice caving mining. This study defines the internal and external fields of the overburden strata in oblique working faces and reveals the evolutionary characteristics of the overburden structure when the lower slice caving face passes through residual section coal pillars.

**Fig. 18:**
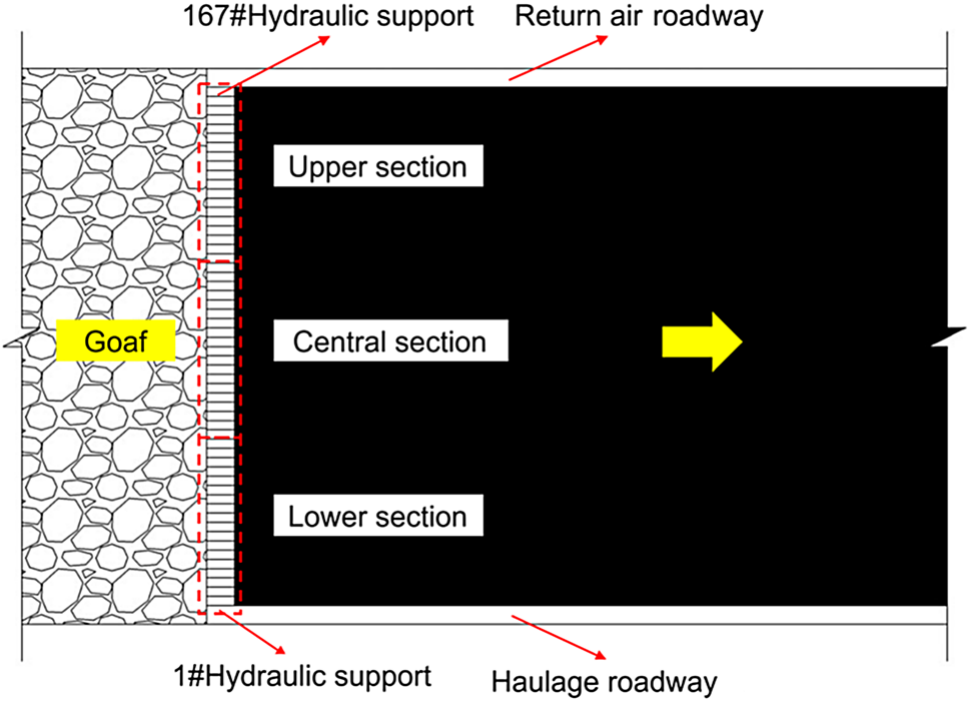
Hydraulic support layout in the working face.

To further investigate abutment pressure variations during lower slice mining through oblique sectional coal pillars, a total of 167 sets of ZFY14500/25/42D two-leg shield hydraulic supports were installed in the lower slice working face 250,303 at Yanbei Coal Mine, with a centre distance of 1.75 m. For every 5 fully mechanised hydraulic supports, a KJ653-F2 mine-use intrinsically safe roof pressure wireless monitoring substation was installed on the support column. Simultaneously, a YN60SZ mechanical mine pressure observation meter was installed on each support. The layout of the hydraulic supports on the working face is illustrated in Fig. 18. During the advancement of the working face, the position of the monitoring station and working resistance of the support were monitored and saved, and data analysis was performed to dynamically monitor the mine pressure manifestation characteristics of the working face.

**Fig. 19:**
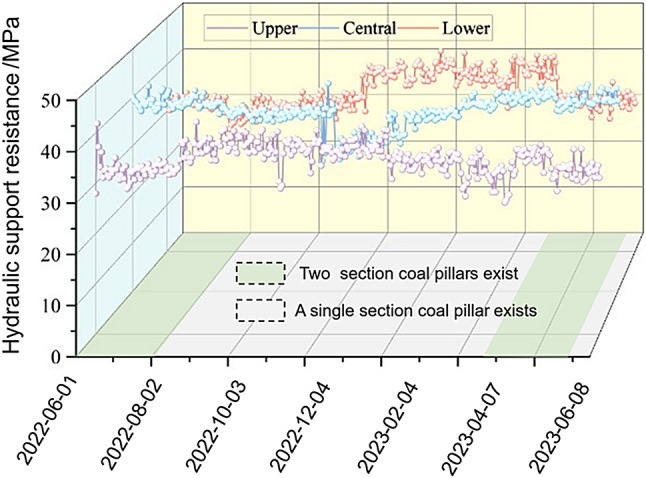
Working resistance of the hydraulic support in the working face.

Through on-site measurements of the 250,303 lower working face of the Yanbei Coal Mine, the working resistance of the support for a complete section of the coal pillar from June 2022 to June 2023, and the average working resistances of the upper, middle, and lower parts of the working face were obtained. The lower support of the lower sliced working face was 1–56# support, the central support of the working face was 57–112# support, and the upper support of the working face was 113–167# support. The specific characteristics are shown in Fig. 19.

When section coal pillars were present in the roof strata, pillar instability caused the original abutment pressure to transfer laterally to both sides of the face. The overlying strata experienced first-time fracturing with relatively low caving heights, resulting in hydraulic support pressures ranging between 30 and 36 MPa. In contrast, goaf areas exhibit large-scale roof caving and debris accumulation, leading to significantly higher support pressures of 36–42 MPa. Field monitoring data of shield resistance quantitatively validated the spatial correlation between pressure distribution and residual pillar locations. The observed patterns showed remarkable consistency with both the physical similarity simulation results and support resistance characteristics recorded in the lower slice of working face 250,203 from June 2022 to June 2023. Consequently, the advancement of the 250,203 lower working face requires enhanced support measures to be promptly implemented when the immediate roof consists of upper-slice goaf areas. Simultaneously, close monitoring of the hydraulic support conditions is essential to prevent structural component failure and hydraulic system damage.

Mining activities in the lower slice caving face induce instability in the upper slice residual coal pillars, triggering a large-scale overburden collapse. Within the disturbance zone, the roof structure progressively evolved, forming a composite structure consisting of lower-position inverted-step combination cantilever beams and a high-position Voussoir beam structure. As the intersection angle between the lower slice caving face and the overlying residual coal pillar varied, the fracture pattern of the external field overburden exhibited an approximately symmetric trapezoidal shape, while the dynamic instability of the structure led to an evolutionary process of internal field collapse morphology through asymmetric double-arch, symmetric double-arch, and single-arch configurations. These findings provide significant guidance for the safe mining of thick coal seams with split-level caving faces.

The identified “crescent-shaped stress zone” beneath residual pillars (Fig. 16) provides a basis for optimizing support design. For example, hydraulic supports in the central section should be rated for 46–48 MPa to withstand peak stress, while lateral sections can use 38–42 MPa supports.

The evolutionary sequence of overburden structures (asymmetric double-arch → symmetric double-arch → single-arch) guides the timing of roof control measures. Preemptive hydraulic fracturing of key strata is recommended when the working face is 15–20 m from the pillar edge to mitigate dynamic instability.

Unlike horizontal residual pillars in Western coalfields, the oblique arrangement in Chinese ultra-thick seams introduces unique stress anisotropy. Our findings offer a framework for adapting split-level caving methods to similar geological conditions (e.g., the Powder River Basin, USA, and the Kuznetsk Basin, Russia).

Future investigations will implement discrete element modeling (DEM) to characterize the transitional behavior between continuum and discrete fracture regimes in stratified top-coal caving systems. The proposed methodology will: (1) simulate fracture network evolution under 20° oblique excavation conditions using bonded-particle models (BPM) with resolution ≤ 0.5 m; (2) quantify progressive failure mechanisms in residual inter-slice pillars through a newly developed multi-parameter failure criterion incorporating stress anisotropy (K = σ₁/σ₃≥2.5) and damage accumulation (D ≥ 0.7); and (3) validate predictions against in-situ microseismic monitoring data from the Yanbei Mine case study.

## Conclusions

1. During the advancement of the lower-slice working face, periodic passage through the upper-slice residual section pillars induces a cyclic evolution in the roof failure range: expanding from the influence of two upper-slice goafs to three, then contracting back to two. This cyclic pattern is directly linked to the oblique intersection (20°) between the lower-slice face and upper-slice pillars, as the gradual lateral shift of pillar influence zones prolongs the disturbance duration compared to vertical or parallel layouts.
2. The vertical stress distribution in residual pillars exhibits distinct phasic characteristics: prior to lower-slice mining, stress concentration is approximately symmetrical with a peak of 39.2–42.5 MPa. When the working face is 15–45 m from the pillar center during entry and exit, the stress peaks at 45.3 MPa (13.7% increase) and 46.7 MPa (9.9% increase), respectively, accompanied by significant pillar deformation. Directly beneath the pillar, stress releases in a crescent-shaped pattern, validated by both FLAC^3D^ simulations and field monitoring of hydraulic support resistance.
3. Overburden collapse under oblique pillar conditions is spatially partitioned into internal and external fields: The internal field (secondary fracture zone) evolves dynamically with pillar position, transitioning from asymmetric double-arch (single pillar at face edge) to symmetric double-arch (single pillar at face center), and finally to single-arch (dual pillars at face edges), with vertical displacements ranging from 10.5 to 30 m. The external field (key strata collapse zone) forms a quasi-symmetric trapezoidal structure, expanding from two goaf widths (single pillar) to three goaf widths (dual pillars), constrained by high-positioned voussoir beam structures.
4. Mining-induced instability of residual pillars triggers large-scale overburden collapse, driving the roof structure to evolve into a composite bearing system: lower-position inverted-step cantilever beams (formed by secondary fracturing of immediate roof) and high-position large-scale voussoir beams (formed by key strata fragmentation). This composite structure redistributes overburden loads, with arch-shaped superimposition of cantilever and voussoir beams providing primary support.
5. Field monitoring confirms that pillar instability transfers abutment pressure laterally, resulting in distinct support resistance characteristics: 30–34 MPa under intact pillars (first-time fracturing of overlying strata) and 38–42 MPa under goaf areas (large-scale gangue accumulation). These findings necessitate zone-specific support design, with central sections requiring higher-rated hydraulic supports (46–48 MPa) to withstand peak stresses.

## Data Availability

The data sets generated in this study are not publicly available. These data form part of the current research project. We must wait until this research project is completed before we can make all data public. The data are available from the corresponding author upon request.
